# Master protocols in low- and middle-income countries: a review of current use, limitations and opportunities for precision medicine

**DOI:** 10.1136/bmjgh-2024-018561

**Published:** 2025-07-25

**Authors:** Luke O Ouma, Sarah Al-Ashmori, Samuel Sarkodie, Lou Whitehead, Ann Breeze Konkoth, Shaun Hiu, Theophile Bigirumurame, Dorcas Njeri Kareithi, Jingky Lozano-Kuehne, Marzieh Shahmandi, James M S Wason

**Affiliations:** 1 Biostatistics research group, Newcastle University Faculty of Medical Sciences, Newcastle upon Tyne, UK; 2 Nuffield Department of Primary Care Health Sciences, University of Oxford, Oxford, UK

**Keywords:** Global Health, Clinical trial, Randomised control trial, Review

## Abstract

**Background:**

Master protocols—umbrella, basket and platform trials, study multiple therapies, multiple diseases or both, offering many advantages, most profoundly that they answer multiple treatment-related questions, which would otherwise take multiple trials. We conducted a review of clinical trial registries to characterise their use in advancing precision medicine in low and middle-income countries (LMICs).

**Methods:**

We searched trial records available in 20 trial registries globally, including ClinicalTrials.gov and WHO ICTRP, to identify umbrella, basket and platform trials launched until 30 September 2023.

**Results:**

We identified 102 master protocols—29 umbrella trials, 31 basket trials, 36 platform trials, as well as six other designs that partially aligned with the working definition of master protocols, run in 54 different LMICs. Most trials were pharmaceutical industry-sponsored studies (60/102, 58.8%), conducted in oncology settings (56/102, 54.9%), currently ongoing (69/102, 67.6%) in early phase (phase I and II) settings (70/102, 68.6%) and have been planned or launched in the last 5 years (93/102, 91.2%), mainly with international collaborations in high-income countries. China was a site to more than half of all master protocols (53/102, 52%), and only a small proportion of trials (5/102, 4.9%) launched exclusively in LMICs excluding China and European middle-income countries. For most studies, aspects of trial design and trial documentation (including study protocols and analysis plans) were not publicly accessible.

**Conclusion:**

Unlike high-income countries, where several hundreds of master protocols are ongoing or completed, there is limited use of master protocols in LMICs, partly owing to low penetration of precision medicine research and limited clinical trial infrastructure in most LMICs. The evidence presented here creates a case for supporting precision medicine initiatives in LMICs (especially Africa) and training and capacity building initiatives focused on innovative clinical trial designs like master protocols, especially in therapeutic areas outside oncology.

**WHAT IS ALREADY KNOWN ON THIS TOPIC**

- Master protocols enable simultaneous evaluation of multiple interventions or subpopulations under a single overarching framework, now predominantly used in oncology and in high-income countries.
- Master protocols enable simultaneous evaluation of multiple interventions or subpopulations under a single overarching framework, now predominantly used in oncology and in high-income countries.

**WHAT THIS STUDY ADDS**

- This study provides a comprehensive review of the use of master protocols in LMICs, highlighting their low penetration and identifying China and European middle-income countries as notable exceptions.
- It underscores the need for broader adoption of these trial designs in LMICs, particularly in therapeutic areas beyond oncology, and emphasises the critical role of precision medicine initiatives and robust trial infrastructure in supporting such advancements.

**HOW THIS STUDY MIGHT AFFECT RESEARCH, PRACTICE OR POLICY**

- Policymakers and stakeholders in global health may be encouraged to prioritise training and capacity-building efforts in innovative clinical trial designs.
- Expanding the use of master protocols could improve the efficiency and relevance of clinical trials in LMICs, ultimately enhancing healthcare equity and access to precision medicine advancements.

## Introduction

 Master protocols, comprising umbrella, basket and platform trials, have revolutionised the drug development landscape in many therapeutic areas by allowing the study of multiple therapies, multiple diseases or both under a single trial infrastructure.^1^ These trial designs advance the cause of precision medicine where the goal is to tailor treatments based on patient-specific factors such as genetics, biomarkers, disease subtypes or other treatment-related characteristics. Basket trials evaluate single or combination therapies in several diseases that share a common characteristic,^1^ such as a biomarker or disease pathway. Umbrella trials allow concurrent investigation of multiple targeted therapies within a single disease setting.^2^ Platform trials are similar to umbrella trials, except they adaptively allow therapies to enter and leave the trial. Given the known worldwide variation in causes, drivers, clinical presentations and pathogenesis of many diseases, so are the strategies to prevent and treat them. As such, innovative designs are of high relevance in advancing global health, especially where resource constraints often hinder traditional trial designs, and we need to identify effective treatments by maximising efficiency and reducing costs.^3^,^7^

The advantages of master protocols have been well characterised, including operational efficiency, logistical advantages, faster recruitment through centralised screening and simultaneous approval of substudies, all contributing to accelerated drug development.^1 8 9^ Today, these designs have been advantageous in settings including COVID-19,^3^ cancer,^4 5^ immune-mediated inflammatory diseases^6^ and Alzheimer’s disease,^7^ by accelerating the speed of drug trials. Basket trials, for instance, enable inclusion of rare diseases and can rapidly identify responsive patient populations, exemplified by imatinib’s approval for multiple rare and life-threatening cancers from a single-arm basket trial.^10^ The flexible and adaptive framework in umbrella and platform trials can reduce trial duration, resources and ultimately cost by eliminating the need for separate trials. The RECOVERY trial during the COVID-19 pandemic demonstrated this by conclusively assessing at least 10 experimental therapies in under 2 years.^11^ However, practical challenges exist, such as treatment response heterogeneity in basket trials which can complicate data interpretation and analysis and the need for well-defined biomarker-driven stratification in umbrella trials. For platform trials, their complexity as well as need for long-term infrastructure can pose significant hurdles. Nevertheless, the ability of master protocols to expedite drug development and refine precision medicine strategies arguably makes them indispensable in modern drug development.

A high-level examination of the geographic distribution of master protocol use reveals that the rise in master protocol use in the last decade is mostly confined to high-income countries, usually involving early phase trials in oncology. Park *et al,*^12^ in their 2019 review, reported that only 6/83 master protocols (VE-BASKET,^13^ FUTURE,^14^ TRUMP,^15^ EBOLA,^16^ GBM AGILE,^17^ DIAN-TU^7^) had an LMIC country representation. Together, these six studies spanned only three LMICs: China, Brazil and Mexico. In other systematic reviews of master protocols,^18 19^ the geographic coverage of master protocols by country was not characterised. Furthermore, the quantity of clinical trial research in LMICs remains inadequate, despite experiencing the greatest burden of disease globally.^20 21^ The greater disease burden in LMICs, rapidly changing healthcare needs and the need for timely evaluation of promising (drug and non-drug) interventions (as in the EBOLA or COVID-19 pandemic) arguably justify the need to design and deliver innovative clinical trials across a broad range of diseases and treatment types in LMICs. Currently, the rarity of master protocols in LMICs can be attributed in part to the focus on published studies and limited awareness of their utility beyond oncology and biomarker-driven studies. Besides, master protocols arguably face greater operational and regulatory challenges,^22^ especially in regions with limited trial infrastructure such as LMICs, thus limiting their use to settings such as China and European middle-income countries where trial infrastructure is fairly well-developed. 

In this paper, we present a review of clinical trial registries that seeks to unravel a clearer representation of the current landscape, including all studies that have been planned, launched and/or stopped early. Through a clearer understanding of the status quo, we develop a case for improving the use of master protocols in LMICs across various diseases, reflecting on some concepts, potential barriers, opportunities for their use and a way forward.

## Methods

### Data sources and search strategy

We conducted a search of registered clinical trials present in 20 different trial registries across the world, that provide information to the WHO International Clinical Trials Registry Platform (ICTRP).^23^ Our search included trial records registered since 01 July 2005 (enforcement of mandatory trial registration by International Committee of Medical Journal Editors (ICJME)) up to 30 September 2023. A full list of these registries is provided in online supplemental appendix S1, including Clinicaltrials.gov,^24^ Pan African Clinical Trials Registry^25^ and EU clinical trials register.^26^ A comprehensive search (see online supplemental material) was run in clinicaltrials.gov and WHO ICTRP. In addition, we conducted manual forward/backward citation checks of relevant trials from recent reviews of master protocols^12 18 19 27^ including one done by our group.^2^

### Inclusion criteria

We included registered trials conducted in at least one or more sites in a LMIC country, according to the World Bank income classification of countries.^28^ Given the common mislabelling of the different master protocols in the literature, we adopted the standardised definitions as provided in online supplemental table S1 and recategorised the trials if they were misclassified according to our working definitions. When a master protocol did not neatly fit the definitions of either umbrella, basket or platform trial—by combining elements of more than one master protocol—it was included and categorised as a complex design. All trial records were independently assessed for inclusion by two reviewers, and conflicts regarding the relevance of a record were resolved by a third reviewer.

### Data extraction and synthesis

Each individual trial record was independently extracted for information by two reviewers. We extracted information on trial identifiers (trial ID and acronyms), master protocol type, trial phase, disease area, the number of modules (subtrials in an umbrella design; disease subgroups in a basket trial; arms including the control in a platform trial), the sample size, randomisation, error rate control, the primary outcome, any adaptive features, the analysis approach (Bayesian or frequentist), trial sponsor, countries covered, recruitment and completion status, among other considerations. A listing of all information extracted from the trial registry is available in the (online supplemental table S2). The data were then synthesised into a single dataset, from which the descriptive analysis was undertaken.

### Patient and public involvement

This study did not involve patients and/or the public in its design, conduct, reporting or dissemination.

## Results

We retrieved a total of 10 281 trial records across the various registries from our initial search. After removal of duplicates and records not meeting eligibility criteria, 189 trial records of 102 unique master protocol trials were included in our study. We found 31 basket trials, 29 umbrella trials, 36 platform trials and six complex master protocol designs conducted in 54 countries. A complete list of all trial records is available in online supplemental material.

### Trends of master protocols in LMICs

**Figure 1:**
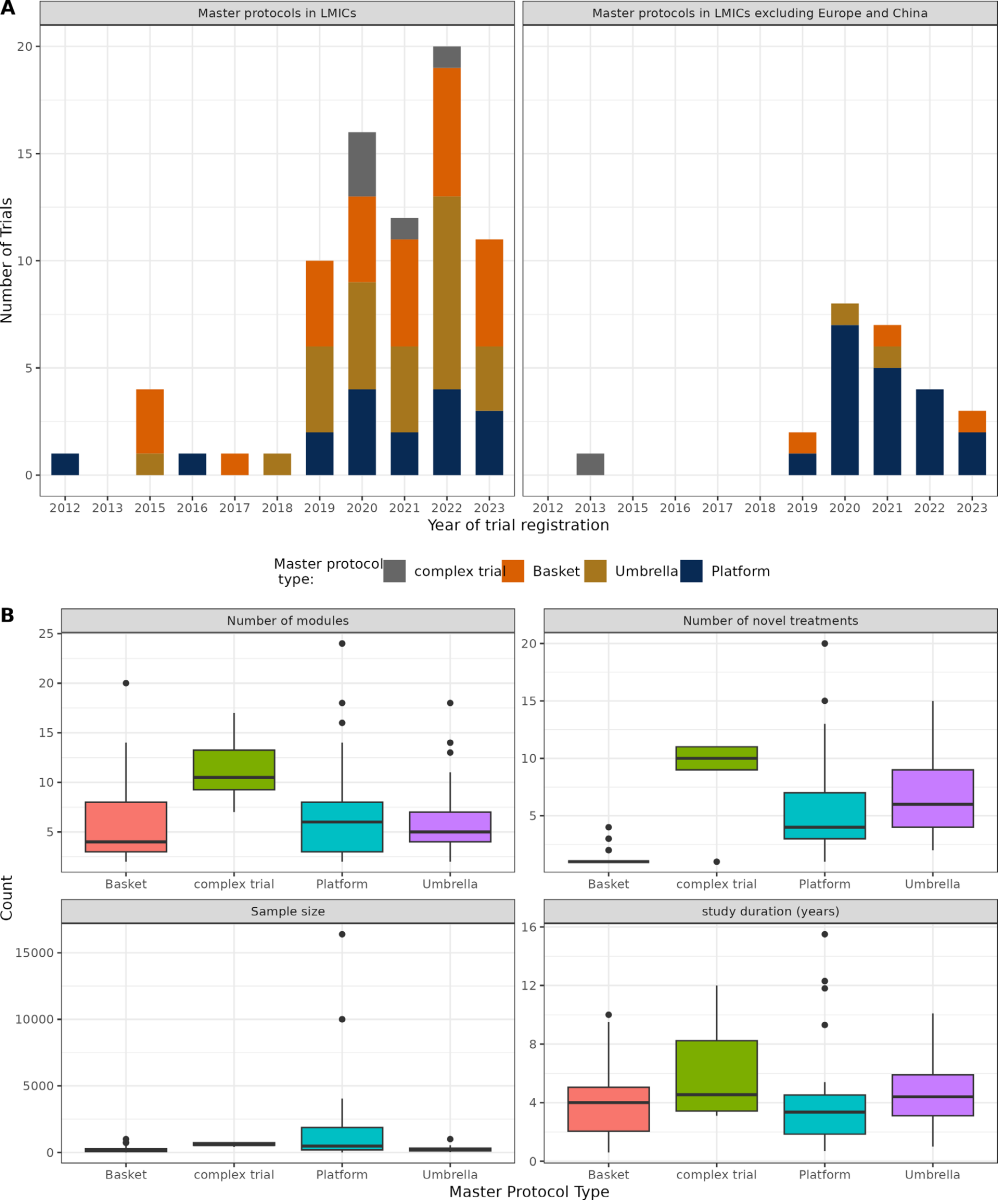
Trends and descriptive characteristics of master protocols in LMICs. (**A**) Trends of master protocols in LMICs over time. The statistics here show new trial registrations within a particular year. (**B**) Summary of novel treatments, modules, sample size and study duration of the master protocols in LMICs. LMICs, low- and middle-income countries.

Master protocols have been gaining popularity in LMICs, particularly over the last 5 years (2019 onwards) (figure 1A). This is consistent with the increasing interest in running trials in these regions, especially post pandemic (online supplemental figure S1), although their inclusion in global multisite trials is still low (online supplemental figure S2). Notably, the majority of the master protocol trials reported in this review (93/102, 91.2%) were launched after the onset of the COVID-19 pandemic. The earliest master protocol reported in our study was the DIAN-TU^6^ platform trial in Alzheimer’s disease that started in 2012 and ran across 13 countries, 3 of which were LMICs (Argentina, Colombia and Mexico). The earliest master protocol that ran exclusively in LMIC sites was TAC^29^ in 2015, an umbrella trial in hepatitis C virus-infected patients in West and Central Africa.

Our results show that most master protocols in LMICs are run in China (n=52), European middle-income countries like Turkey (n=18) and Russia (n=16) and upper middle-income countries such as Brazil (n=22), Argentina (n=16), Mexico (n=14), South Africa (n=12), India (n=7) and Colombia (n=6). In online supplemental figure S3, we observe that, while the majority of trials were multicountry studies, the proportion of LMICs was often less than 30%, figures that were lower when not considering China and LMICs in Europe. For instance, nearly half (48/102, 47.1%) of the trials did not have an LMIC site outside of China and Europe. Excluding European middle-income countries and China, there are far fewer master protocol trials ongoing in other LMIC regions. The majority of these were platform trials (19/102, 18.6%) followed by basket (3/102, 2.9%) and then umbrella trials (2/102, 1.9%). 

### Characteristics of master protocols in LMICs

We present a summary of the master protocol trial characteristics in table 1. Oncology was the most common therapeutic area (54.9%, 56/102) where master protocols are conducted/ongoing, mainly featuring umbrella and basket trials. There was an increase in the uptake of master protocols in both oncology and non-oncology areas over time. Between 2012 and 2018, we identified 2.9% of master protocols (3/102) in oncology and 3.9% (4/102) in non-oncology. From 2019 to 2023, this increased significantly, with 54.9% (56/102) in oncology and 45.1% (46/102) in non-oncology. Thus, the aforementioned trend in the rise of master protocols in LMICs was consistently reflected across both oncology and non-oncology fields. Notably, the most common disease area used in non-oncology was COVID-19 and other respiratory diseases. Platform trials were the most common master protocol design used in non-oncology (28/46, 60.9%), followed by basket trials (14/46, 30.4%).

**Table 1:** Trial characteristics of identified master protocols in LMICs

Variable	Basket (n=31)		Umbrella (n=29)		Platform trials (n=36)		Complex designs*	Total (N=102)
	LMIC incl.	LMIC excl.	LMIC incl.	LMIC excl.	LMIC incl.	LMIC excl.		
Disease setting								
Oncology	16 (51.6%)	1 (3.2%)	23 (79.3%)	2 (6.9%)	7 (19.4%)	1 (2.8%)	6 (100.0%)	56 (54.9%)
Non-oncology	12 (38.7%)	2 (6.5%)	4 (13.8%)	0 (0.0%)	10 (27.8%)	18 (50.0%)	0 (0.0%)	46 (45.1%)
Trial phase								
Phase I	2 (6.5%)	0 (0.0%)	1 (3.4%)	0 (0.0%)	1 (2.8%)	0 (0.0%)	0 (0.0%)	4 (3.9%)
Phase II	14 (45.2%)	1 (3.2%)	14 (48.3%)	1 (3.4%)	5 (13.9%)	6 (16.7%)	5 (83.3%)	46 (45.1%)
Phase I/II	3 (9.7%)	0 (0.0%)	11 (37.9%)	1 (3.4%)	3 (8.3%)	2 (5.6%)	0 (0.0%)	20 (19.6%)
Phase III	7 (22.6%)	1 (3.2%)	0 (0.0%)	0 (0.0%)	3 (8.3%)	6 (16.7%)	0 (0.0%)	17 (16.7%)
Phase II/III	0 (0.0%)	0 (0.0%)	0 (0.0%)	0 (0.0%)	3 (8.3%)	3 (8.3%)	0 (0.0%)	6 (5.9%)
Phase IV	0 (0.0%)	0 (0.0%)	1 (3.4%)	0 (0.0%)	1 (2.8%)	2 (5.6%)	0 (0.0%)	4 (3.9%)
Unspecified	2 (6.5%)	1 (3.2%)	0 (0.0%)	0 (0.0%)	1 (2.8%)	0 (0.0%)	1 (16.7%)	5 (4.9%)
Study sponsor								
Industry-funded	24 (77.4%)	1 (3.2%)	14 (48.3%)	2 (6.9%)	8 (22.2%)	9 (25.0%)	4 (66.7%)	60 (58.8%)
Government funded	4 (12.9%)	2 (6.5%)	13 (44.8%)	0 (0.0%)	9 (25.0%)	10 (27.8%)	2 (33.3%)	42 (41.2%)
Sponsor category								
Pharma	23 (74.2%)	1 (3.2%)	13 (44.8%)	2 (6.9%)	8 (22.2%)	5 (13.9%)	4 (66.7%)	56 (55.0%)
Academia	1 (3.2%)	1 (3.2%)	8 (27.6%)	0 (0.0%)	6 (16.7%)	8 (22.2%)	1 (16.7%)	26 (25.5%)
Government research institutes	1 (3.2%)	0 (0.0%)	1 (3.4%)	0 (0.0%)	2 (5.6%)	2 (5.6%)	0 (0.0%)	6 (5.9%)
Hospitals	2 (6.5%)	0 (0.0%)	4 (13.8%)	0 (0.0%)	1 (2.8%)	0 (0.0%)	1 (16.7%)	8 (7.8%)
Non-profit organisation	0 (0.0%)	0 (0.0%)	1 (3.4%)	0 (0.0%)	0 (0.0%)	2 (5.6%)	0 (0.0%)	3 (2.9%)
Investigator sponsored	1 (3.2%)	0 (0.0%)	0 (0.0%)	0 (0.0%)	0 (0.0%)	2 (5.6%)	0 (0.0%)	3 (2.9%)
Completion status								
Completed	2 (6.5%)	0 (0.0%)	1 (3.4%)	0 (0.0%)	2 (5.6%)	6 (16.7%)	0 (0.0%)	11 (10.8%)
Not started	1 (3.2%)	1 (3.2%)	4 (13.8%)	0 (0.0%)	1 (2.8%)	0 (0.0%)	0 (0.0%)	7 (6.8%)
Ongoing	18 (58.1%)	2 (6.5%)	21 (72.4%)	2 (6.9%)	12 (33.3%)	10 (27.8%)	4 (66.7%)	69 (67.7%)
Suspended/terminated	4 (12.9%)	0 (0.0%)	1 (3.4%)	0 (0.0%)	2 (5.6%)	3 (8.3%)	1 (16.7%)	11 (10.8%)
Unknown	3 (9.7%)	0 (0.0%)	0 (0.0%)	0 (0.0%)	0 (0.0%)	0 (0.0%)	1 (16.7%)	7 (6.8%)
Study population								
Adolescents	1 (3.2%)	0 (0.0%)	0 (0.0%)	0 (0.0%)	0 (0.0%)	0 (0.0%)	0 (0.0%)	1 (1.0%)
Adults	20 (64.5%)	3 (9.7%)	27 (93.1%)	2 (6.9%)	15 (41.7%)	16 (44.4%)	5 (83.3%)	88 (86.3%)
Adolescents adults	1 (3.2%)	0 (0.0%)	0 (0.0%)	0 (0.0%)	0 (0.0%)	3 (8.3%)	0 (0.0%)	4 (3.9%)
Children adolescents	2 (6.5%)	0 (0.0%)	0 (0.0%)	0 (0.0%)	1 (2.8%)	0 (0.0%)	0 (0.0%)	3 (2.9%)
All	4 (12.9%)	0 (0.0%)	0 (0.0%)	0 (0.0%)	1 (2.8%)	0 (0.0%)	1 (16.7%)	6 (5.9%)
Eligible sex								
Female	1 (3.2%)	0 (0.0%)	9 (31.0%)	0 (0.0%)	3 (8.3%)	0 (0.0%)	0 (0.0%)	13 (12.7%)
Both	27 (87.1%)	3 (9.7%)	18 (62.1%)	2 (6.9%)	14 (38.9%)	19 (52.8%)	6 (100.0%)	89 (87.3%)
Type of interventions								
Pharmaceutical	26 (83.9%)	2 (6.5%)	26 (89.7%)	2 (6.9%)	16 (44.4%)	19 (52.8%)	6 (100.0%)	97 (95.1%)
Non-pharmaceutical	2 (6.5%)	1 (3.2%)	0 (0.0%)	0 (0.0%)	1 (2.8%)	0 (0.0%)	0 (0.0%)	4 (3.9%)
Both	0 (0.0%)	0 (0.0%)	1 (3.4%)	0 (0.0%)	0 (0.0%)	0 (0.0%)	0 (0.0%)	1 (1.0%)
Study published								
Yes	4 (13.0%)	0 (0.0%)	4 (13.8%)	0 (0.0%)	6 (16.7%)	9 (25.0%)	2 (32.3%)	25 (24.5%)
No	24 (77.4%)	3 (9.7%)	23 (79.2%)	2 (6.8%)	11 (30.6%)	11 (27.7%)	4 (66.7%)	77 (75.5%)
Published material								
Study protocol	1 (3.2%)	0 (0.0%)	0 (0.0%)	0 (0.0%)	0 (0.0%)	2 (5.6%)	2 (32.3%)	5 (4.9%)
Study design	0 (0.0%)	0 (0.0%)	2 (6.9%)	0 (0.0%)	1 (2.8%)	0 (0.0%)	0 (0.0%)	3 (2.9%)
Results	3 (9.6%)	0 (0.0%)	1 (3.4%)	0 (0.0%)	4 (11.1%)	5 (13.9%)	0 (0.0%)	13 (12.8%)
Study design and results	0 (0.0%)	0 (0.0%)	1 (3.4%)	0 (0.0%)	1 (2.8%)	2 (5.6%)	0 (0.0%)	4 (3.9%)
None	24 (77.4%)	3 (9.7%)	23 (79.3%)	2 (6.9%)	11 (30.6%)	10 (27.8%)	4 (66.7%)	77 (75.5%)
Trial documents								
Study protocol	1 (3.2%)	0 (0.0%)	0 (0.0%)	0 (0.0%)	2 (5.6%)	3 (8.3%)	1 (16.7%)	7 (6.9%)
Study protocol SAP	2 (6.4%)	0 (0.0%)	0 (0.0%)	0 (0.0%)	1 (2.8%)	1 (2.8%)	0 (0.0%)	4 (3.9%)
None	25 (80.6%)	3 (9.7%)	27 (93.1%)	2 (6.9%)	14 (38.9%)	15 (41.7%)	5 (83.3%)	91 (89.2%)

LMIC excl. refers to the category of trials run exclusively in LMIC countries.

LMIC incl. refers to the category of trials run in at least one LMIC country.

* Complex designs refer to trial designs that do not neatly fit the working definitions adopted in supplementary material section 1.

LMIC, low- and middle-income country; SAP, statistical analysis plan.

Most of the master protocols (67.7%) are still ongoing, with just 11/102 studies (10.8%) having been completed. A small proportion of trials (10.8%) have either been withdrawn, suspended or terminated, and seven studies (6.8%) registered on a trial database as of September 2023 were yet to start. In terms of trial phases, the majority of master protocols were early phase studies (70%) (ie, phase I or phase II studies), compared with late phase studies. While umbrella, basket and platform designs were equally common in early-phase studies, late-phase studies were often dominated by platform trials. 

Our findings further show that the master protocols were commonly industry-sponsored studies (58.8%) investigating pharmaceutical interventions (95.1%) and conducted in adult populations (≥18 years old; 86.3%), enrolling both sexes (87.3%). Trial documentation, such as study protocol and statistical analysis plans (SAPs), was mostly unavailable (89.2%), and only a few studies had been published (75.5%), due to the smaller proportion of completed studies.

### Design considerations of the master protocols 

We present the trial design and statistical properties for the master protocols in figure 1 and online supplemental table S3. Of the 102 studies, randomisation was fairly common (57.8%), followed by adaptive design features (62.7%) and a frequentist approach to trial design (75.5%). The most common adaptive features were adding and dropping of arms (23.5%), often a feature of platform trials and seamless designs (22.5%), that is, phase I/II and phase II/III studies, followed by group sequential designs (7.8%) and dose adaptations (7.8%). These adaptive design features, including adaptive randomisation and sample-size re-estimation, were commonly present in master protocols. Seamless designs were commonly present in umbrella and platform trials and rarely in basket studies, partly attributed to the several treatments under investigation in umbrella and platform designs. Bayesian approaches to trial design were less common overall (13.7%) but were more commonly used in platform trials (10/36, 27.8%). 

However, in more than one-third of trial records (37.3%), it was unclear whether any adaptive features were present, largely attributed to the widespread unavailability of relevant trial documentation such as SAPs and trial protocols in trial databases.

Complex designs had on average higher number of novel treatments under investigation (median=10), the highest number of subtrials/modules (median=10.5), larger sample sizes (median=596) and longer trial duration (median=5 months) compared with the conventional master protocols like umbrella, platform and basket trials (figure 1B).

In terms of the conventional master protocols, platform trials, on average, had the highest number of subtrials/modules (median=6) and recruited the largest sample size (median=477) when compared with umbrella and basket trials. However, they had a shorter study duration (median=3.4 years) and included fewer novel treatments (median=4) compared with umbrella trials. This is unsurprising for platform trials given their varied use across different therapeutic areas and their common use of adaptive design.

### Geographical distribution of master protocols in LMICs 

**Figure 2:**
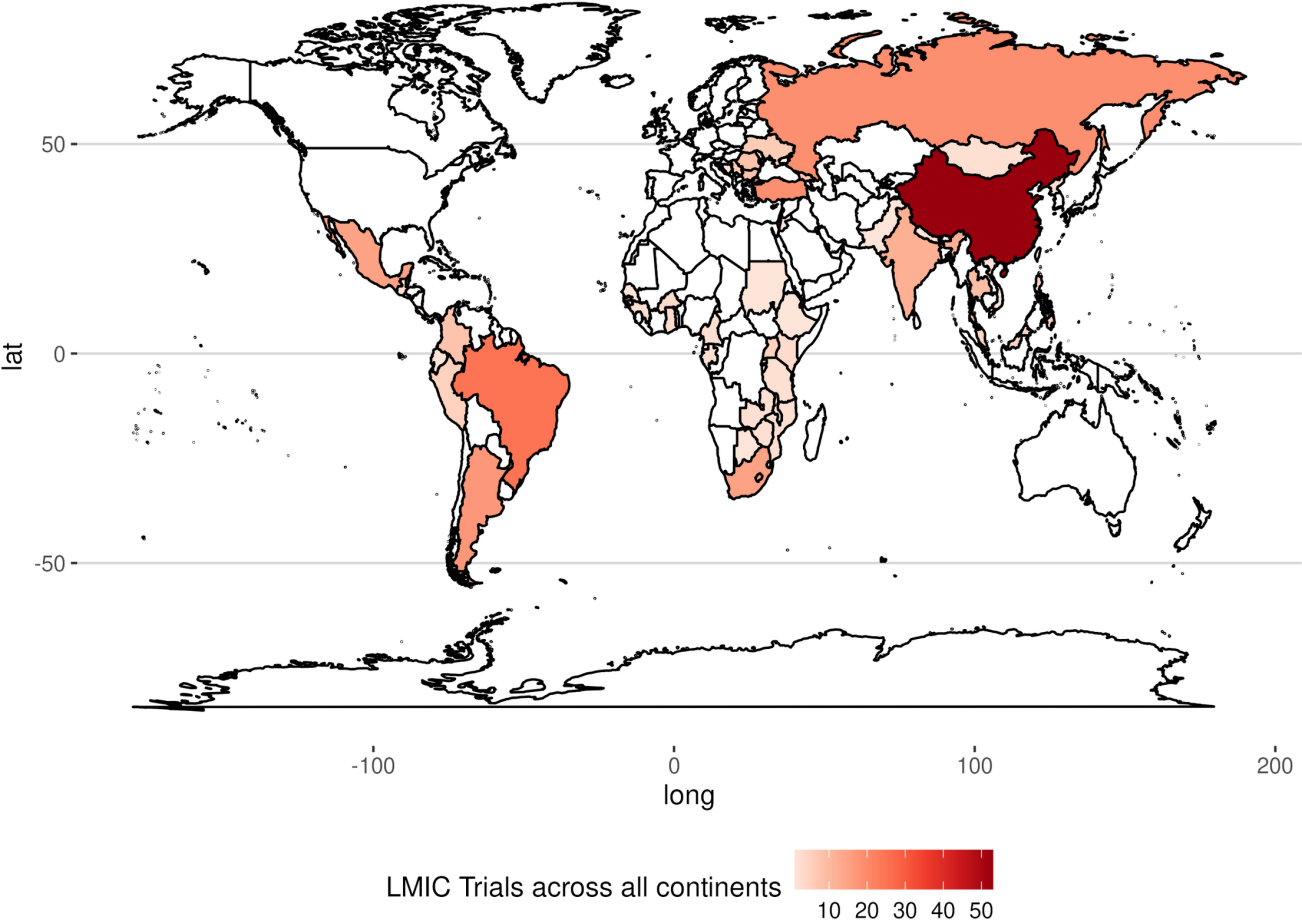
Geographical representation of master protocols in LMICs. The map is generated from data in our review. Dense colours represent a higher number of master protocols in those regions. LMICs, low- and middle-income countries.

We present the geographical distribution of the master protocols in figure 2 and online supplemental table S4. Table 2 highlights classical examples of these trials, with the entire list available in online supplemental table S5. Master protocols in this review were mostly multicountry trials (65.7%), with a greater representation from China, Brazil and Russia. In Online supplemental figure S5, we illustrate geographical disparities in how master protocol utilisation varies by population size, seeing that some of the most populous countries like China and Brazil are common study sites. There were only five studies conducted exclusively in LMIC outside China and Europe, and these were three platform studies on COVID-19, a single platform study on tuberculosis and one multibasket study on Human epidermal growth factor receptor 2 (HER2)/epidermal growth factor receptor (EGFR) solid tumours. Of these, four out of five studies were conducted exclusively in Africa, except for one study on COVID-19 (online supplemental material section 2.2). One trial on HER2/EGFR solid tumours was terminated, while the rest are currently ongoing. Thus, as of November 2024, no trial evaluating targeted therapies under a master protocol framework has been designed, run and completed in Africa to the best of our knowledge.

**Table 2:** Highlighted examples of ongoing or completed master protocol trials in LMICs

Trial ID/registration	Trial design	Disease setting	Description
EUCTR2020-0 04 457–76	Basket trial	Crohn’s disease, ulcerative colitis and juvenile psoriatic arthritis	A phase 3, multicentre, open-label, basket, long-term extension study of Ustekinumab in paediatric clinical study participants aged 2–18 years across 13 countries.
ACTG A5288	Umbrella trial	HIV	An open-label phase IV study of HIV-1 infected participants with triple-class experience or resistance to nucleoside reverse transcriptase inhibitors (NRTIs), non-NRTIs and protease inhibitors and who were failing their current regimen. Patients were assigned to one of four cohorts (A, B, C or D) based on their screening genotype results and antiretroviral history where novel agents are investigated in each cohort.
ANTICOV PACTR 2020–06537901307	Platform trial	COVID-19	An open-label, randomised, adaptive platform trial testing the safety and efficacy of treatments in mild-to-moderate COVID-19 patients. The trial aimed to identify early treatments that could prevent progression of COVID-19 to severe disease and potentially limit transmission
NCT04589845	Complex trial	Solid tumours	A phase II, global, multicentre, open-label, multicohort study to evaluate the safety and efficacy of targeted therapies or immunotherapy in participants with unresectable, locally advanced or metastatic solid tumours that harbour specific oncogenic genomic alterations or who are tumour mutational burden-high, as identified by a validated next generation sequencing assay.

LMICs, low- and middle-income countries.

Regionally, most studies were conducted in Asia, South America and Europe (82.3%), with a few studies spread across Africa, South Africa accounting for a greater majority. The median number of countries per trial was 7 (IQR: 1–13) overall, and the median number of LMICs per trial was 1. Excluding China and European LMIC representation, the median number of LMICs per trial was only 1. 

## Discussion

Although master protocol clinical trials can efficiently answer multiple research questions over traditional trial designs, LMICs remain under-represented in global research of this type. We reviewed clinical trial registries to understand the actual extent of implementation of master protocols and found increasing implementation of master protocols in LMICs, but they are largely dominated by China and middle-income countries in Europe.

The under-representation of LMICs in master protocol trials is concerning, considering the potential benefits that these designs could offer in these settings where resources for conducting traditional large scale trials are limited. Master protocols could provide an efficient approach to study multiple treatments and indications (including rare diseases), thus optimising resources. The limited use of master protocols outside of China and European middle-income countries highlights the potential existence of unique challenges to the implementation of master protocols in LMICs. In these settings, limited infrastructure and financial resources for managing complex designs, ethical and regulatory system obstacles and operational barriers have been reported as critical barriers to conducting trials in developing countries.^30^ Besides, the limited penetration of precision medicine and related technology in most LMICs^31^ and lack of expertise to design and implement these designs in lower-income countries have further limited their use. 

This review, similar to recent reviews,^12 18 32^ reports a higher prevalence of master protocols in oncology and early phase trial settings. However, of interest to note is the increasing utilisation of master protocols in non-oncology areas. For LMICs, this signals new opportunities to leverage the advantages of master protocols to address important treatment-related questions in infectious and non-communicable diseases that are leading public health concerns in Africa and many LMIC regions. Already, diseases such as HIV, Ebola and COVID-19 have welcomed the use of umbrella and platform trials^2^ and greater opportunities for indications such as tuberculosis and neglected tropical diseases to accelerate investigation of life-saving therapies to meet the needs of patients in resource-poor settings.

The increased awareness and opportunities afforded by master protocols in both oncology and non-oncology settings, however, present both opportunities and challenges for LMICs. To ensure LMICs fully capitalise on this evolving trend, it is imperative that several key considerations are addressed. First, there is an urgent need for capacity-building initiatives for stakeholders, including regulators, researchers and patient advocacy groups. This includes initiatives aimed at strengthening clinical trial infrastructure, training researchers in master protocols design and conduct, improving regulatory frameworks and enhancing data management capabilities. These, we summarise in table 3 alongside potential solutions to enhance their adoption in LMICs. Beyond the LMIC specific challenges, Cecchini *et al*^33^ discuss at depth pragmatic issues experienced by various stakeholders involved with real master protocols. Without these foundational elements, LMICs will continue to lag behind in the global master protocol trials landscape and in implementing their own regionally or locally relevant studies.

**Table 3:** Summary of potential challenges and solutions when running master protocols in LMICs

Challenge	Description	Potential solutions
Complex trial designs	Umbrella, platform and basket trials have multiple arms and objectives, increasing complexity.	Capacity-building initiatives for stakeholders including regulators, researchers and patient advocacy groups. Enhance coordination and streamline communication among stakeholders; implement comprehensive planning upfront.
Regulatory barriers	Complex and inconsistent regulatory frameworks across countries can slow approval and implementation.	Harmonising regulatory requirements through regional cooperation, advocating for streamlined approvals. Collaboration with agencies in HICs like FDA, EMA with extensive expertise in master protocols, to enable knowledge transfer, harmonisation of standards and capacity building.
Patient recruitment and retention	Complex protocols may deter patient participation due to burdensome consent processes and trial demands.	Involve patient advocacy groups early to understand patient perspectives; develop simplified and clear consent forms.
Genomic-related challenges	Limited genomic data from diverse populations; limited local biomarker testing infrastructure.	Establish genomic databases by facilitating local research initiatives and international and industry collaborations for broader data inclusivity.
Ethical considerations	Ensuring ethical standards across multiple sites with varying ethical boards.	Establishing centralised ethics review committees and standardised informed consent procedures.
Infrastructure limitations	Limited clinical trial infrastructure and trained personnel.	Strengthening local clinical trial infrastructure. Establish robust data management systems and frameworks for ethical data sharing and cross-border collaboration.
Limited expertise with master protocols	Shortage of trained personnel in design, management and analysis of master protocols.	Training programmes, knowledge transfer from high-income countries Multicountry master protocols involving HICs and LMICs

EMA, European Medicines Agency; FDA, Food and Drug Agency; HIC, high income country; LMICs, low- and middle-income countries.

Our findings unearth the skewed nature of existing collaborations in recent and completed master protocols. These collaborations are largely concentrated in high-income countries and consistently select LMICs like China and Brazil, and in Africa, primarily South Africa, where clinical trial infrastructure is fairly well established. Moreover, we see that technical expertise behind the design of many master protocols was domiciled in HICs—for example, the statistical expertise behind the design of the EBOLA platform trial^34^ and ANTICOV trial in COVID-19^35^ was all domiciled in HICs. Despite these and known global inequities in precision medicine research,^36^ since master protocols are commonly multicountry studies, we see this as an opportunity for leveraging partnerships that facilitate knowledge transfer, enable capacity-building and provide financial and technical support to overcome the barriers that LMICs currently face.^31 37^ For example, joint research initiatives or public-private partnerships could pool resources and expertise to launch master protocol trials targeting health challenges specific to LMICs. In this way, LMICs could not only benefit from cutting-edge scientific methodologies but also contribute to global health innovation. As a result, LMICs can spearhead their own research in times such as the COVID-19 pandemic, where efficient designs to investigate treatments faster and more efficiently are much needed.

Among the important considerations and enablers for these innovative designs, regulatory frameworks stand out. Here, we reflect on the possibilities of regulatory harmonisation between HICs and LMICs given their flexibilities and complexity over traditional designs. Even in HICs, challenges with regulators are a norm. Researchers have reported varying levels of acceptance of these submissions (particularly in the confirmatory setting) and inconsistent regulatory feedback.^32^ We anticipate the inexperience of agencies in LMICs can pose a considerable challenge to adoption of these trials, being in favour of less complex proposals, hence the need for regional collaborations or capacity-building initiatives.

For master protocols, as a vehicle for precision medicine therapy development, questions about the rationale for precision medicine in LMICs are inevitable. Despite concerns about feasibility, precision medicine in LMICs is rooted in the potential for improved treatment outcomes, reduced adverse effects and more efficient healthcare spending. While critics may argue that precision medicine is resource-intensive and less justifiable in settings with pressing public health needs, emerging evidence suggests that a one-size-fits-all approach to treatment may be less effective and even more costly in the long run. Misra *et al*^38^ highlight how precision approaches can optimise care for non-communicable diseases, a growing burden in LMICs, by tailoring treatments to genetic and environmental risk factors specific to these populations. The application of master protocols in LMICs can thus support the development of locally relevant precision medicine strategies, ensuring that novel therapies are both effective and accessible within these settings. Addressing infrastructural challenges, strengthening biobanking and genomic research and fostering collaborations will be critical in advancing this paradigm.

Our review has a few limitations. First, publicly available clinical trial registry data are often incomplete or inconsistently reported, with variations in registry reporting practices across different regions. Some master protocol trials might not have been explicitly labelled as such, leading to potential underestimation of their prevalence. Additionally, the review protocol was not registered a priori in a database such as PROSPERO, although we adhered to systematic review principles.

In conclusion, while the current landscape of master protocols shows ongoing progress, there is significant potential for broader adoption in LMICs, particularly in non-oncology settings. We argue that there are considerable benefits to be gained in drug development through wider implementation of master protocols in LMIC settings. Strategic efforts aimed at enhancing clinical trial capacity, fostering cross-border collaboration and harmonising regulatory standards will be crucial in realising the full potential of master protocols in LMICs. Such efforts are necessary to ensure that LMICs are not left behind in the global shift towards more innovative and efficient trial designs, ultimately improving the speed at which new treatments reach patients in these regions.

## Supplementary material

10.1136/bmjgh-2024-018561 online supplemental file 1

## Data Availability

All data relevant to the study are included in the article or uploaded as supplementary information.
