# Supplementary material for: Master protocols in low- and middle-income countries: a review of current use, limitations and opportunities for precision medicine
Source: BMJ Glob Health. 2025 Jul 25;10(7):e018561. doi: 10.1136/bmjgh-2024-018561 (PMC12306224; doi:10.1136/bmjgh-2024-018561)
Supplement: online supplemental file 1 [file bmjgh-10-7-s001.pdf]

Supplementary material to "Master protocols in Low-and-Middle income countries: A review of current use, limitations, and opportunities for precision medicine"

Luke Ouma[1], Sarah Al-Ashmori[1], Samuel Sarkodie[1], Lou Whitehead[1], Shaun Hiu[1,2], Ann Breeze Konkoth[1], Theophile Bigirumurame[1], Dorcas Kareithi[1] Jingky Lozano-Kuehne[1], Marzieh Shahmandi[1], James M.S. Wason[1]

1. Population Health Sciences Institute, Newcastle University, Newcastle upon Tyne, UK

2. Nuffield Department of Primary Care Health Sciences, University of Oxford, Oxford, UK

*Corresponding Author : Luke Ouma; Address: Population Health Sciences Institute, Ridley Buildng 1, Richardson Road, Newcastle upon Tyne NE2 4AX, UK;

Telephone number: +44(0)0191-208-6000; Email address: ouma.ondijo@gmail.com

1. Appendix S1: Supplementary information

1.1. Literature search strategy

The following literature search term was used in the trial registries:

`("basket design") OR ("basket study") OR ("basket trial") OR ("matrix approach") OR ("matrix design") OR ("matrix study") OR ("matrix trial") OR ("platform approach") OR ("platform design") OR ("platform study") OR ("platform trial") OR ("umbrella approach") OR ("umbrella design") OR ("umbrella study") OR ("umbrella trial") OR ("master protocol").`

1.2. Data sources

The following list of trial registries provide data to the WHO ICTRP repository, and primary sources of information for master protocols included in our review:

- Australian New Zealand Clinical Trials Registry
- Chinese Clinical Trial Registry
- ClinicalTrials.gov
- Clinical Trials Information System (CTIS)
- EU Clinical Trials Register (EU-CTR)
- International Standard Randomized Controlled Trial Number (ISRCTN)
- The Netherlands National Trial Register
- Brazilian Clinical Trials Registry (ReBec)

- Clinical Trials Registry - India
- Clinical Research Information Service - Republic of Korea
- Cuban Public Registry of Clinical Trials
- German Clinical Trials Register
- Iranian Registry of Clinical Trials
- Japan Registry of Clinical Trials (jRCT)
- Pan African Clinical Trial Registry
- Sri Lanka Clinical Trials Registry
- Thai Clinical Trials Registry (TCTR)
- Peruvian Clinical Trials Registry (REPEC)
- Lebanese Clinical Trials Registry (LBCTR)
- International Traditional Medicine Clinical Trial Registry (ITMCTR)

1.3. Definitions and types of Master protocol trial designs

Table S1: Definitions and types of Master protocol trial designs

**Terminology**	**Definition**
Master protocol	Trial design that evaluates multiple diseases, multiple treatments, or both under a single trial infrastructure and protocol[1].
Basket trial	Evaluates one targeted therapy on multiple diseases or multiple disease subtypes.
Umbrella trials	Evaluates multiple targeted therapies for different subgroups of a single disease[2].
Platform trial	Evaluates multiple experimental treatments in a single disease in a perpetual manner[1].
Complex design	A trial design that combine features of any two or more master protocol trial designs (for instance both umbrella and basket features).

1.4. Data extraction template

We extracted the following information listed in Table S2 for all relevant trials.

Table S2: Data extracted from master protocol trials included in the review

- Trial ID
- Study acronym
- Public & scientific title
- What phase of trial is it (e.g., phase II)?
- Eligible gender
- Eligible age category (e.g child, adult)
- Intervention type (e.g., drug, device, vaccine)
- Disease area &specific disease (e.g., oncology)
- How many modules/arms are there in the design?
- How many novel treatments (i.e., other than standad of care) are investigated?
- Actual/planned sample size
- Master protocol classification (by authors)
- What is/are the primary outcome(s)?
- Available supporting information (e.g., study protocol, SAP)
- Has the study been published? If yes, which information published
- Countries where study is undertaken
- Study sponsor type
- Study start, end dates and duration
- Is the trial published?
- Trial completion status
- Did it use a randomised, single-arm, or mixed design?
- If it is a randomised design, is the control arm the same across modules?
- Is it an adaptive or non-adaptive design?
- Is the analysis pooled or separate?
- Did the sample size calculation use a separate or pooled approach?
- What is the statistical analysis method for the primary outcome?
- Is the analysis Bayesian or frequentist?

2. Appendix S2: Supplementary results

2.1. Trends and characteristics of master protocols

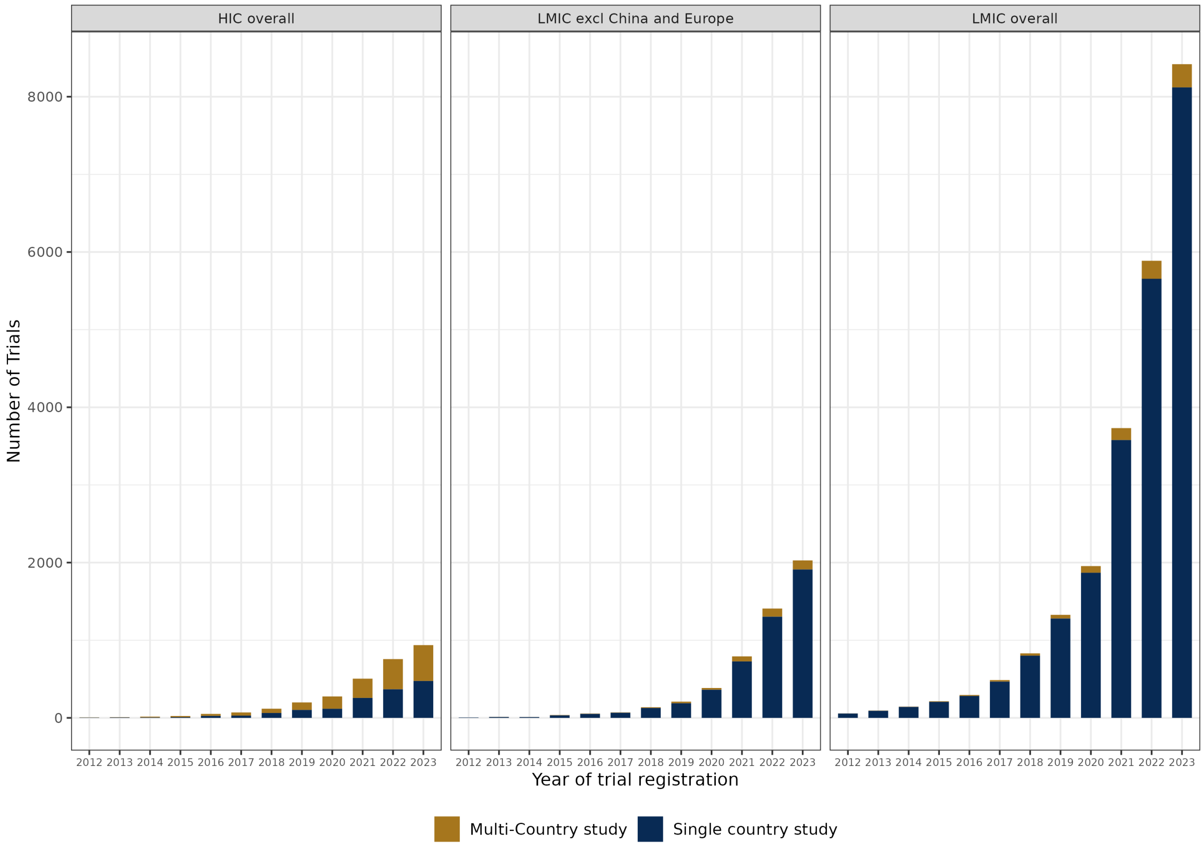

Figure S1: Trends in HIC and LMIC country representation for all trials registered globally between 2012-2023. Data are obtained from clinicaltrials.gov for contrast on LMIC inclusion in global trials .

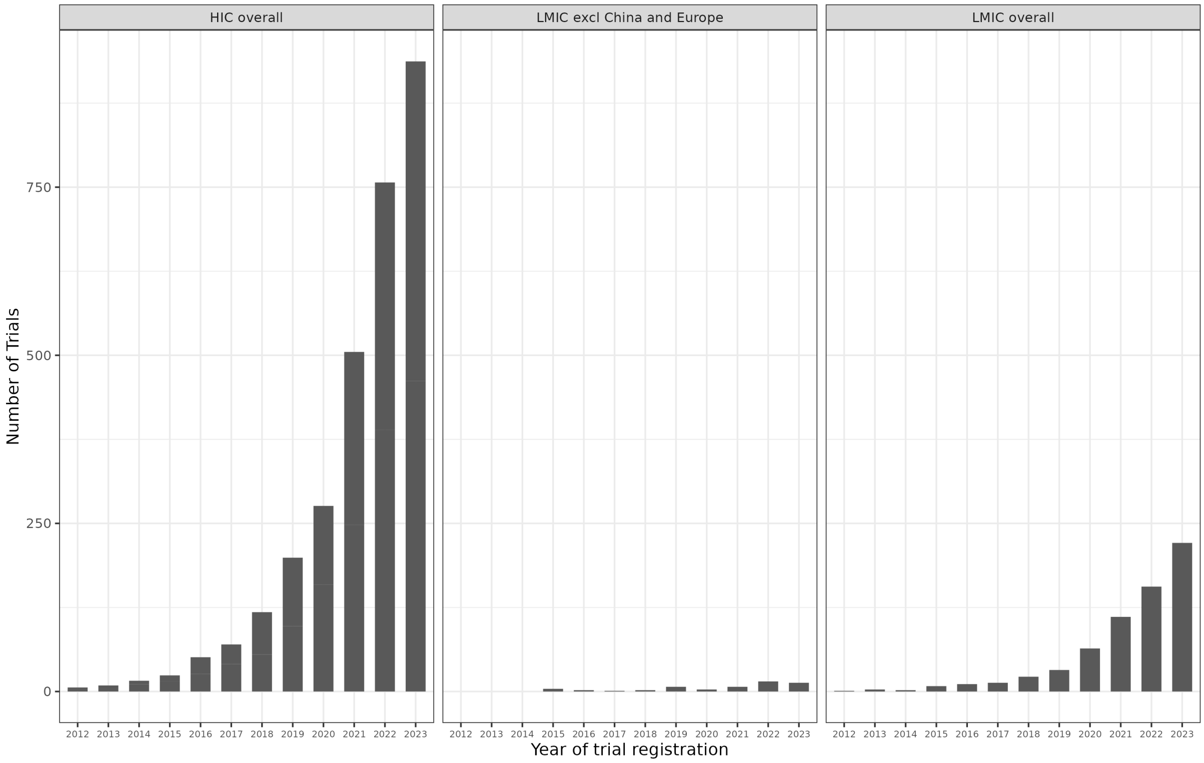

Figure S2: Trends in HIC and LMIC country representation for all trials registered globally between 2012-2023, where LMIC involvement inclusion is at least 30% of all study sites. We compute proportion of LMIC involvement as the number of all LMIC countries in the study as a proportion of total number of country sites.

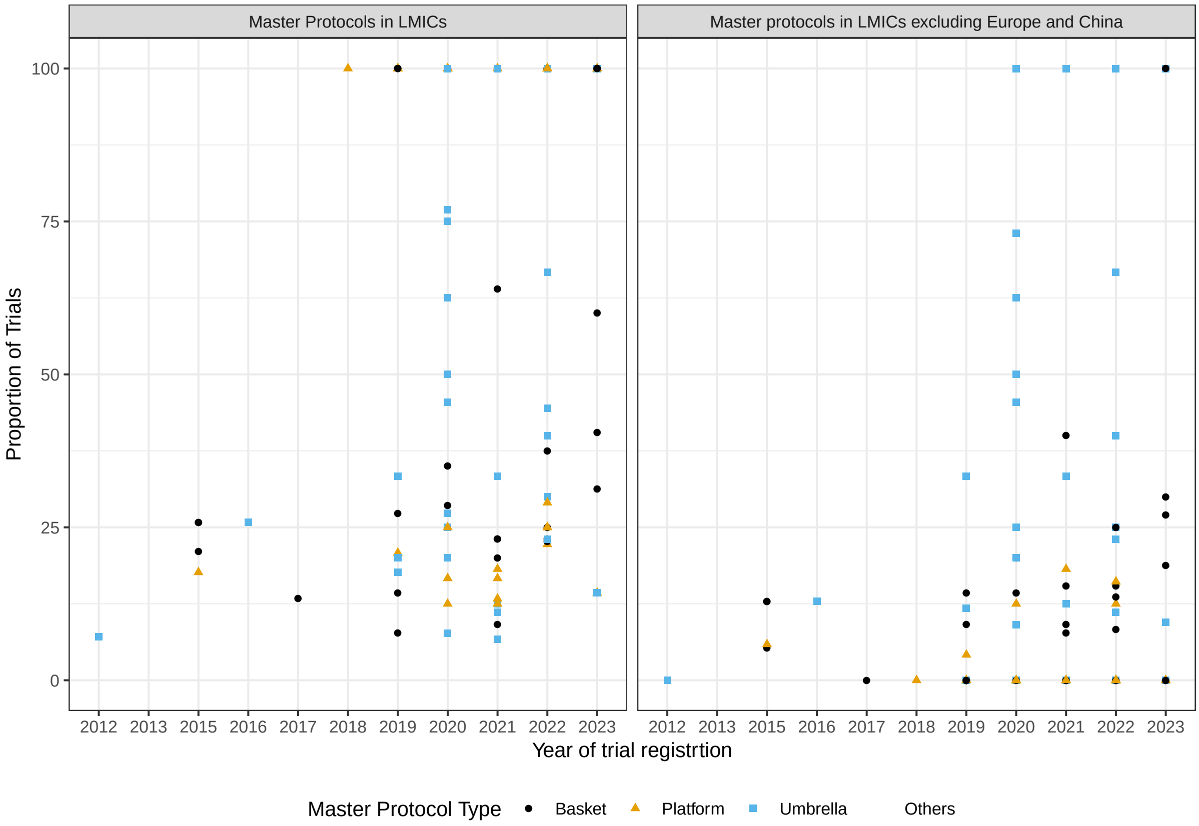

Figure S3: Proportion of LMIC country representation in master protocols. Each point on the graph represents a unique trial included in our review. The proportions are derived as the number of LMIC countries in a specific trial over total number of countries where the trial is run globally.

Table S3: Statistical characteristics of identified Master protocols

Variable	Basket (N=31)		Umbrella (N=29)		Platform (N=36)		Complex designs (N=6)		Total (N=102)
	LMIC incl.	LMIC excl.	LMIC incl.	LMIC excl.	LMIC incl.	LMIC excl.	LMIC incl.	LMIC excl.	
**Randomised**[c]									
Yes	9 (29.0%)	2 (6.5%)	13 (44.8%)	1 (3.4%)	14 (38.9%)	19 (52.8%)	1 (16.7%)	0 (0.0%)	59 (57.8%)
No	19 (61.3%)	1 (3.2%)	14 (48.3%)	1 (3.4%)	3 (8.3%)	0 (0.0%)	4 (66.7%)	1 (16.7%)	43 (42.2%)
**Adaptive design**[c]									
Yes	8 (25.8%)	0 (0.0%)	17 (58.6%)	1 (3.4%)	17 (47.2%)	19 (52.8%)	1 (16.7%)	1 (16.7%)	64 (62.7%)
No	14 (45.2%)	2 (6.5%)	6 (20.7%)	1 (3.4%)	0 (0.0%)	0 (0.0%)	1 (16.7%)	0 (0.0%)	24 (23.5%)
Unclear	6 (19.4%)	1 (3.2%)	4 (13.8%)	0 (0.0%)	0 (0.0%)	0 (0.0%)	3 (50.0%)	0 (0.0%)	14 (13.8%)
**Adaptive design type**[c]									
Adding and dropping arms	1 (3.1%)	0 (0.0%)	11 (36.7%)	1 (3.3%)	17 (47.2%)	19 (52.8%)	0 (0.0%)	0 (0.0%)	46 (44.2%)
Group Sequential	3 (9.4%)	0 (0.0%)	0 (0.0%)	0 (0.0%)	0 (0.0%)	3 (8.3%)	1 (16.7%)	0 (0.0%)	7 (6.7%)
Adaptive randomisation	0 (0.0%)	0 (0.0%)	0 (0.0%)	0 (0.0%)	1 (2.8%)	2 (5.6%)	0 (0.0%)	0 (0.0%)	3 (2.9%)
Adaptive treatment switching	1 (3.1%)	0 (0.0%)	0 (0.0%)	0 (0.0%)	0 (0.0%)	0 (0.0%)	0 (0.0%)	0 (0.0%)	1 (1.0%)
Sample size re-estimation	0 (0.0%)	0 (0.0%)	0 (0.0%)	0 (0.0%)	0 (0.0%)	1 (2.8%)	0 (0.0%)	0 (0.0%)	1 (1.0%)
Seamless design	3 (9.4%)	0 (0.0%)	11 (36.7%)	1 (3.3%)	5 (13.9%)	4 (11.1%)	0 (0.0%)	0 (0.0%)	24 (23.1%)
Dose adaptations	3 (9.4%)	0 (0.0%)	2 (6.7%)	0 (0.0%)	2 (5.6%)	1 (2.8%)	0 (0.0%)	0 (0.0%)	8 (7.7%)
Efficacy/futility stopping	0 (0.0%)	0 (0.0%)	0 (0.0%)	0 (0.0%)	0 (0.0%)	1 (2.8%)	0 (0.0%)	1 (16.7%)	2 (2.0%)
Unclear/None	0 (0.0%)	0 (0.0%)	0 (0.0%)	0 (0.0%)	0 (0.0%)	1 (2.8%)	0 (0.0%)	1 (16.7%)	38 (37.3%)
**Bayesian/Freq. design**[c]									
Bayesian	1 (3.2%)	0 (0.0%)	3 (10.3%)	0 (0.0%)	3 (8.4%)	7 (19.5%)	0 (0.0%)	0 (0.0%)	14 (13.7%)
Frequentist	24 (77.5%)	3 (9.7%)	21 (72.4%)	2 (6.9%)	11 (30.6%)	9 (25.0%)	4 (66.7%)	1 (16.7%)	77 (75.5%)
Unclear/unspecified	3 (9.7%)	0 (0.0%)	3 (10.3%)	0 (0.0%)	3 (8.3%)	1 (2.8%)	1 (16.7%)	0 (0.0%)	11 (10.8%)
**Pooled analysis**[c]									
Yes	3 (9.7%)	0 (0.0%)	2 (6.9%)	0 (0.0%)	0 (0.0%)	1 (2.8%)	3 (50.0%)	0 (0.0%)	9 (8.8%)
No	8 (25.8%)	1 (3.2%)	11 (37.9%)	0 (0.0%)	8 (22.2%)	4 (11.1%)	1 (16.7%)	1 (16.7%)	34 (33.3%)
Unclear/unspecified	17 (54.8%)	2 (6.5%)	14 (48.3%)	2 (6.9%)	9 (25.0%)	13 (38.9%)	1 (16.7%)	0 (0.0%)	59 (57.9%)
**Common control**[c]									
Yes	9 (29.0%)	0 (0.0%)	10 (34.5%)	1 (3.4%)	12 (33.3%)	18 (50.0%)	0 (0.0%)	0 (0.0%)	50 (49.0%)
No	19 (61.3%)	3 (9.7%)	17 (58.6%)	1 (3.4%)	5 (13.9%)	1 (2.8%)	5 (83.3%)	1 (16.7%)	52 (51.0%)

2.2. Geographical characteristics of identified Master protocols

Table S4: Geographical distribution of Master protocol trials in LMICs

Variable	Basket	Umbrella	Platform	Total
Single/Multi-country[c]				
Multi-country	23 (69.7%)	9 (37.5%)	35 (77.7%)	67 (65.7%)
Single country	10 (30.3%)	15 (62.5%)	10 (22.3%)	35 (34.3%)
Geographical region				
LMI [1]	0 (0.0%)	0 (0.0%)	2 (4.4%)	2 (2.0%)
UMI [2]	13 (39.4%)	4 (16.7%)	20 (44.4%)	37 (36.3%)
LI [3]; LMI; UMI	0 (0.0%)	0 (0.0%)	3 (6.7%)	3 (2.9%)
LMI; UMI	2 (6.1%)	0 (0.0%)	2 (4.4%)	4 (3.9%)
Unspecified	18 (54.5%)	20 (83.3%)	18 (40.1%)	56 (54.9%)
Countries per trial (median)	12	1	8	21
LMICs per trial (median)	2	1	2	5
LMICs per trial excl euro/china (median)	1	1	1	3

[1]Lower Middle Income; [2]Upper Middle Income; [3]Low Income

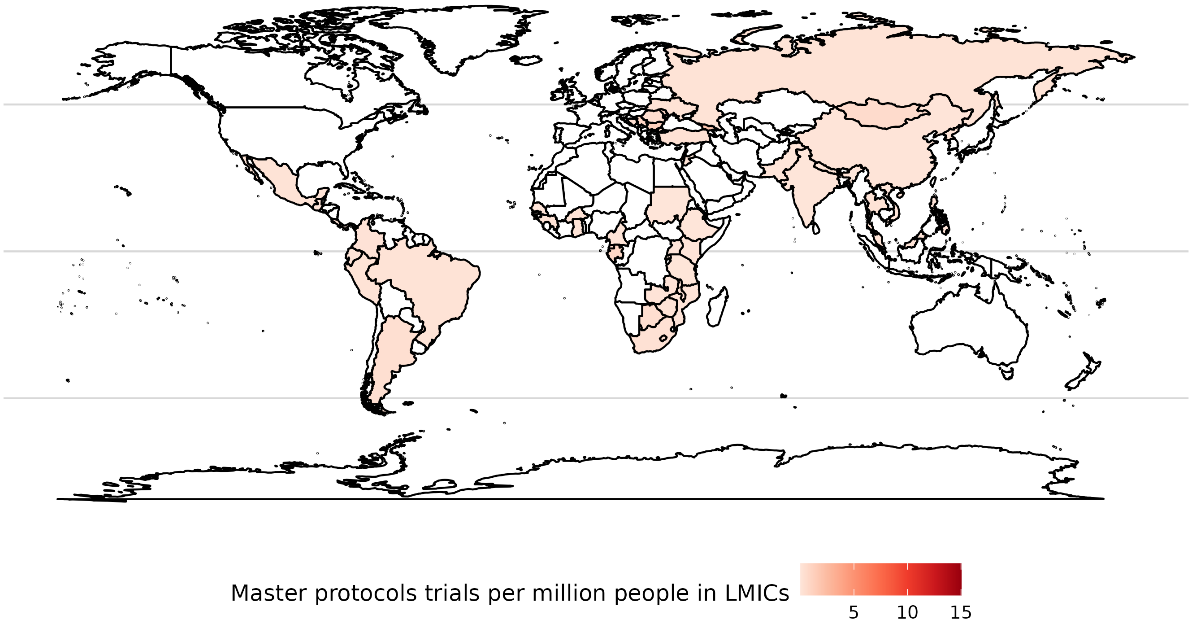

Figure S4: Geographic distribution of master protocols conducted in LMICs, normalised by population size. The depth of colours show increasing number of trials per million people. We utilise the latest World Bank 2023 population figures.

3. Appendix S3: List of included studies

Table S5: List of master protocols in LMICs included in the review

**TrialID**	**No. countries overall**	LMIC countries	Classification	Trial phase	Disease setting
EUCTR2015-000269-30-DE	19	China;Turkey;Russia;Brazil	Basket	Phase 2	Cancer
EUCTR2017-000387-14-FR	13	Turkey	Basket	Phase 2	Obesity
EUCTR2020-002829-28-NL	9	Turkey;Russia	Umbrella	Phase 1/2	Cancer
EUCTR2020-004457-76-FR; EUCTR2020-004457-76-PL	13	Turkey;Russia;Argentina	Basket	Phase 3	Crohn Disease; Ulcerative Colitis; Juvenile Psoriatic Arthritis
EUCTR2020-004873-29-IT	11	Korea, Democratic Republic	Basket	Phase 2	Cancer
EUCTR2020-005114-18-DE	12	Turkey;Russia	Umbrella	Phase 2	Cancer
EUCTR2020-005386-13-IT	8	Korea, Democratic Republic	Platform	Phase 1/2	Cancer
EUCTR2020-005963-29-DK;ACTRN12620000445976	4	India	Platform	Phase 3	COVID-19
EUCTR2021-002395-39-AT;EUCTR2021-002395-39-DK; EUCTR2021-002395-39-FR; NCT05503264; JPRN-jRCT2011220016	12	Argentina;Russia;China	Basket	Phase 3	Autoimmune Encephalitis
EUCTR2021-005115-32-FI; NCT05565378; EUCTR2021-005115-32-GR	20	Thailand;Turkey; Mexico; Argentina; Brazil;South Africa	Platform	Phase 2	Cancer
ISRCTN13336322	13	Georgia	Platform	Unspecified	respiratory tract infection
ISRCTN27106947	2	South Africa	Platform	Phase 1/2	COVID-19
NCT01760005	14	Serbia	Platform	Phase 2/3	Alzheimers Disease; Dementia
NCT01953926	2	Cameroon;Senegal	Multi-basket	Phase 2	Cancer
NCT02405013	17	Georgia; China; Peru	Umbrella	Phase 2	Hepatitis C
NCT02568267	31	Argentina; Brazil; China; Colombia; India; Russia; Turkey; Ukraine	Basket	Phase 2	Cancer
NCT02576431;CTRI/2021/07/034739	31	Argentina; Brazil; China; Colombia; India; Russia; Turkey; Ukraine	Basket	Phase 2	Cancer
NCT02735707; EUCTR2015-002340-14-NL; CTRI/2021/03/032293	31	Serbia; Bosnia and Herzegovina; Romania; Georgia; Colombia; India; Nepal; Pakistan	Platform	Phase 3	Pneumonia
NCT03386721	15	Russia; Turkey	Basket	Phase 2	Cancer
NCT03574402	1	China	Umbrella	Phase 2	Cancer
NCT03805399	1	China	Umbrella	Phase 1/2	Cancer
NCT03834220	22	Brazil; Bulgaria; Philippines; Romania; Russia; Ukraine	Basket	Phase 2	Cancer
NCT03963622	7	Argentina	Basket	Unspecified	Respiratory Distress Syndrome; Covid-19
NCT03983226	1	China	Umbrella	Phase 2	Cancer
NCT03999658	1	China	Basket	Phase 2	Cancer
NCT04097821; LBCTR2022035001; EUCTR2019-000373-23-DE; EUCTR2019-000373-23-DK	24	Lebanon; Turkey; Russia; Romania; China	Umbrella	Phase 1/2	Myelofibrosis
NCT04126200;EUCTR2019-001138-32-SE	17	Russia; Mexico; Brazil	Platform	Phase 1/2	Cancer
NCT04145193	5	China	Platform	Phase 2	Cancer
NCT04147195	3	Argentina	Platform	Phase 2	Non-alcoholic Fatty Liver Disease

Table 5 (continued..): List of master protocols in LMICs included in the review

**TrialID**	**No. countries overall**	LMIC countries	Classification	Trial phase	Disease setting
NCT04179656	1	China	Basket	Phase 2	Cancer
NCT04215003	1	China	Umbrella	Phase 1/2	Cancer
NCT04221451; JPRN-jRCT2021200023	14	Argentina; Brazil; Russia; Turkey	Basket	Phase 3	Tay-Sachs; Sandhoff
NCT04225715	11	Bulgaria; China; Thailand	Platform	Phase 2	Hepatitis B
NCT04305054; NCT04700072	8	South Africa	Umbrella	Phase 1/2	Cancer
NCT04333732; PACTR202101794986980	11	Ghana; South Africa; Zambia; Uganda; Zimbabwe	Platform	Phase 3	COVID-19
NCT04355858	1	China	Umbrella	Phase 2	Cancer
NCT04395989	1	China	Umbrella	Phase 2	Cancer
NCT04402255	1	Turkey	Basket	Unspecified	Behcet Disease; Familial mediterranean fever
NCT04423185	1	China	Multi-Basket	Phase 2	Cancer
NCT04447755; PER-033-20	20	Argentina; Guatemala; Peru; Russia; Serbia; South Africa;Turkey	Basket	Phase 2	Cancer
NCT04463771	8	Georgia;China	Umbrella	Phase 2	Cancer
NCT04498962	1	China	Basket	Unspecified	Chronic Stable Angina; Vascular Dementia; Idiopathic Membranous Nephropathy
NCT04505774; EUCTR2020-004285-19-ES	5	Brazil	Platform	Phase 4	COVID-19
NCT04518410;CTRI/2021/05/033740; CTRI/2022/01/039426; PER-084-20	26	Argentina; Botswana; Brazil; Colombia; Costa Rica; Dominica; Ecuador; Guatemala; Haiti; India; Kenya; Malawi; Mexico;Peru; Philippines; South Africa;Uganda; Ukraine; Zambia; Zimbabwe	Platform	Phase 2/3	COVID-19
NCT04524871	6	China	Umbrella	Phase 1/2	Cancer
NCT04584008	1	China	All-in-one	Unspecified	Cancer
NCT04589845;JPRN-jRCT2031210670	22	Brazil;China	All-in-one	Phase 2	Cancer
NCT04590586	8	Argentina; Brazil; Mexico; Russia; South Africa;Ukraine	Platform	Phase 3	COVID-19
NCT04593940	8	Jamaica; Argentina; Brazil; Mexico;Peru	Platform	Phase 3	COVID-19
NCT04605562	1	China	Umbrella	Phase 2	Cancer
NCT04810611	8	China	Umbrella	Phase 1	Myelodysplastic Syndromes
NCT04891133	15	Turkey	Platform	Phase 2/3	COVID-19
NCT04903197	9	China	Platform	Phase 1	Cancer
NCT04920838	2	Burkina Faso;Guinea	Platform	Phase 2/3	COVID-19
NCT04931342; EUCTR2020-004936-72-CZ; EUCTR2020-004936-72-DE; EUCTR2020-004936-72-IT	15	Turkey;Russia	Umbrella	Phase 2	Cancer
NCT04955626	6	Brazil;South Africa	Platform	Phase 3	COVID-19
NCT04965519	1	China	Basket	Phase 2	Cancer
NCT04988087	5	China	Basket	Phase 2	Sjogren Syndrome; Mixed Connective Tissue Disease

Table 5 (continued..): List of master protocols in LMICs included in the review

TrialID	No. countries overall	LMIC countries	Classification	Trial phase	Disease setting
NCT05007106; EUCTR2021-001009-56-DE; EUCTR2021-001009-56-NL; EUCTR2021-001009-56-PL	14	Colombia;Turkey	Multi-basket	Phase 2	Cancer
NCT05041907	2	Brazil;Thailand	Platform	Phase 2	COVID-19
NCT05044871	1	China	Umbrella	Phase 2	Cancer
NCT05087381	1	Thailand	Platform	Phase 4	COVID-19
NCT05104567; NCT05061420; EUCTR2021-002150-91-ES; NCT05179603	11	Mexico;Argentina	Umbrella	Phase 2	Cancer
NCT05123482	13	Mexico;China;Thailand	Basket	Phase 1/2	Cancer
NCT05162586	25	Georgia; India; Jordan; Argentina; Brazil; Bulgaria; China; Mongolia; Colombia; Mauritius; Mexico; Moldova; Philippines; Romania; Serbia; South Africa	Basket	Phase 2	Systemic Lupus Erythematosus
NCT05251948	1	China	Umbrella	Phase 1/2	Cancer
NCT05293665	3	Panama;Philippines	Platform	Phase 3	COVID-20
NCT05319730	8	Brazil;China	Umbrella	Phase 1/2	Cancer
NCT05325866	26	India; Argentina; Brazil; Bulgaria; Mexico; Romania	Basket	Phase 1	Cancer
NCT05342636; JPRN-Jrct2031220197	31	Brazil; Bulgaria; China; India; Malaysia; Mexico; Russia; Serbia; South Africa	Umbrella	Phase 1/2	Cancer
NCT05412004; JPRN-jRCT2031220155	8	Brazil;China;Mexico	Basket	Phase 3	Obesity
NCT05458297; EUCTR2021-004450-36-DE; EUCTR2021-004450-36-PT	22	Turkey; Russia; Brazil; Malaysia;Peru	Basket	Phase 2	Cancer
NCT05489211	13	China;Turkey	Multi-basket	Phase 2	Cancer
NCT05549557	1	China	Basket	Phase 1	Cancer
NCT05552157	13	Argentina;Colombia;Mexico	Platform	Phase 2/3	Alzheimers Disease; Dementia
NCT05559008	1	China	Umbrella	Phase 1/2	Cancer
NCT05577702	1	China	Umbrella	Phase 2	Cancer
NCT05582499	1	China	Platform	Phase 1/2	Cancer
NCT05593770	5	Brazil;South Africa	Platform	Phase 2/3	COVID-19
NCT05594095	1	China	Platform	Phase 2	Cancer
NCT05608785	1	China	Umbrella	Phase 1/2	Cancer
NCT05612581	9	Bulgaria; Moldova; Romania; Thailand	Platform	Phase 1/2	Hepatitis B
NCT05635708	4	China	Umbrella	Phase 2	Cancer
NCT05642780	1	China	Basket	Phase 2	Cancer
NCT05648448	1	Thailand	Platform	Phase 2	Influenza
NCT05658692	1	China	Umbrella	Phase 4	Respiratory Distress Syndrome
NCT05682326	37	Argentina; Brazil; Bulgaria; Colombia; India; Malaysia; Mexico; Philippines; Romania; Russia; South Africa; Thailand;Turkey; Ukraine; Vietnam	Basket	Phase 3	Anaemia
NCT05723835; JPRN-Jrct2031220263; CTRI/2023/03/050330	32	Brazil; Bulgaria; China;India; Malaysia; Mexico; Russia; Serbia; South Africa;Thailand	Basket	Phase 3	Turner Syndrome; Noonan Syndrome
NCT05745623	1	China	Basket	Phase 1/2	Cancer
NCT05749588	1	China	Platform	Phase 2	Cancer
NCT05750628; PACTR202302602361661	6	Burkina Faso; Gabon; Ghana; Kenya; Rwanda;Uganda	Platform	Phase 2	Malaria

Table 5 (continued..): List of master protocols in LMICs included in the review

**TrialID**	**No. countries overall**	LMIC countries	Classification	Trial phase	Disease setting
NCT05775159	7	China	Umbrella	Phase 2	Cancer
NCT05807399	6	Gabon; Malawi; Mozambique; South Africa; Tanzania;Uganda	Platform	Phase 2	Tuberculosis
NCT05855408	1	China	Platform	Phase 4	COVID-19
NCT05923073	21	Georgia;India;Brazil	Platform	Phase 3	crohn Disease
NCT05924256	1	China	Umbrella	Phase 2	Cancer
NCT05928780	1	China	Umbrella	Phase 1/2	Cancer
NCT05929079	10	Georgia; India; China;Mongolia; Mexico;Romania	Basket	Phase 3	Diabetes; Obesity
NCT05932173	1	China	Basket	Phase 1/2	Cancer
PACTR202006537901307	12	Burkina Faso; Cameroon; Congo; Equatorial Guinea; Ethiopia; Ghana; Guinea; Kenya; Mozambique; Sudan; Tanzania; Uganda	Platform	Phase 3	COVID-19
PACTR202007700757139	1	South Africa	Platform	Phase 2	COVID-19
RBR-10242sbz	1	Brazil	Basket	Phase 3	oral mucosal diseases

References

1. Woodcock J, LaVange LM. Master Protocols to Study Multiple Therapies, Multiple Diseases, or Both. New England Journal of Medicine. 2017 7;377(1):62-70.

2. Ouma L, Wason J, Zheng H, Wilson N, Grayling M. Design and analysis of umbrella trials: Where do we stand? Frontiers in Medicine. 2022;9.
